# The Effects of Weather on the Flight of an Invasive Bark Beetle, *Pityophthorus juglandis*

**DOI:** 10.3390/insects11030156

**Published:** 2020-03-01

**Authors:** Yigen Chen, Brian H. Aukema, Steven J. Seybold

**Affiliations:** 1 Department of Entomology and Nematology, University of California, Davis, CA 95618, USA; 2 E&J Gallo Winery, 600 Yosemite Blvd., Modesto, CA 95354, USA; 3 Department of Entomology, University of Minnesota, St. Paul, MN 55108, USA; bhaukema@umn.edu; 4 USDA Forest Service Pacific Southwest Research Station, Davis, CA 95618, USA; sjseybold@gmail.com

**Keywords:** climate, humidity, invasive species, *Juglans nigra*, regression analysis, Scolytidae, temperature, thousand cankers disease, walnut twig beetle, weather

## Abstract

The walnut twig beetle, *Pityophthorus juglandis* Blackman (Coleoptera: Scolytidae), vectors the fungus *Geosmithia morbida*, which has been implicated in thousand cankers disease of walnut. Little is known about the flight behavior of the insect across seasons, or about the variability in its flight patterns with weekly fluctuations in weather. We sampled flying adults weekly over a 142-week period (from 29 August, 2011 to 2 June, 2014) with 12-unit black plastic multiple funnel traps baited with a male-produced aggregation pheromone in California, USA. Up to 5000 beetles were captured per trap per week, although catches in most weeks were less than 100 insects. Trap catches were regressed against terms for precipitation, solar radiation, vapor pressure, air temperature, relative humidity, wind speed, and trap catches in preceding weeks. The number of beetles captured in each of the preceding two weeks explained most variation in a current week’s catch. This strong temporal autocorrelation was present in regression models developed for males, females, and both sexes pooled. These models were improved by including two environmental variables. Captures of *P. juglandis* increased with mean weekly air temperature and decreased with increasing mean minimum relative humidity. The percentage of variation in male, female, or total trap catch explained by the temporal variables and the two environmental variables in these multiple regression models ranged from 72% to 76%. While the flight of this invasive insect will likely be affected by site-specific factors as it spreads to new areas, the strong temporal correlation present in this system may provide a useful starting point for developing flight models for newly invaded areas.

## 1. Introduction

The walnut twig beetle, *Pityophthorus juglandis* Blackman (Coleoptera: Scolytidae), is an invasive pest that is native to the southwestern USA and Mexico [1,2]. It has expanded its distribution and occurs in nine western and seven eastern states in the USA [3,4,5,6] and in northern and central Italy [7,8,9]. Together with its symbiotic fungus, *Geosmithia morbida* M. Kolařík, E. Freeland, C. Utley, and N. Tisserat sp. nov. (Ascomycota: Hypocreales) [10], *P. juglandis* causes crown decline and mortality of eastern black walnut trees, *Juglans nigra* L., and other species of walnut and related trees [11,12,13]. Population genetic analyses have revealed two lineages of the beetle, one of which appears to be highly invasive and less genetically diverse [6].

Modelling and predicting flight activity based on abiotic environmental factors is important for elucidating population dynamics and developing pest monitoring techniques for an integrated pest management program. Previous studies have focused on crepuscular flight activity of *P. juglandis* [14,15,16], with Chen and Seybold [16] showing that ambient temperature, light intensity, wind speed, and barometric pressure collectively and interactively affected flight. Furthermore, temperature and barometric pressure individually affected bihourly *P. juglandis* flight as a Gaussian response distribution, with the most flight occurring between 21 and 35 °C and 753 and 761 mbar, respectively. Light intensity and wind speed also individually affected bihourly *P. juglandis* flight in an exponential decay manner, with most flight occurring at less than 4000 lux and less than 5 km/h, respectively. When the four weather variables were considered collectively and interactively, Chen and Seybold [16] concluded that most *P. juglandis* flight occurred at 1) temperatures of ca. 26–27 °C; 2) light intensities of less than 2000 lux; 3) barometric pressures around 755–757 mbar; and 4) wind speeds between 1 and 4 km/h. *Pityophthorus juglandis* is a weak flyer, and the maximum flight distance on a laboratory flight mill without the intervention of wind is approximately 3.6 km in 24 h [17]. In this laboratory assay, very few individuals attained this distance.

To date, the potential association of changes in precipitation, relative humidity, and solar radiation with *P. juglandis* flight is unknown. Periods of rainfall tend to suppress bark beetle flight [18,19]. Changes in humidity can also affect flight behavior, especially for small-bodied insects [19,20,21,22,23]. For instance, the flight distance of an aphid, *Aphis glycines* (Matsumura) (Hemiptera: Aphididae) increased as relative humidity increased from 30% to 75% [23]. Solar radiation encompasses the total frequency spectrum of electromagnetic radiation produced by the sun, covering visible light and near-visible radiation, such as x-rays, ultraviolet radiation, infrared radiation, and radio waves. Although solar radiation and temperature are often correlated positively, flight of the honey bee, *Apis mellifera ligustica* Spinola (Hymenoptera: Apidae), is affected differentially by each factor [24]. These investigators showed that flight increases with increasing temperatures, but may be affected positively or negatively by solar radiation. Some insects and related arthropods are sensitive to particular types of solar radiation. Spider mites, *Tetranychus urticae* Koch (Acari: Tetranychidae) hide on the lower leaf surfaces when radiation from solar UV-B rays (280–315 nm) is strong [25].

Many previous studies investigating associations between flight activity and weather have examined individual or restricted sets of variables [13,26,27]. Temperature is frequently the variable most utilized in previous analyses, given its importance governing life processes for poikilotherms [28]. We have found, however, that the estimate of temperature at which peak *P. juglandis* flight may occur differs slightly between studies [15,16]. Differences in habitats or lengths of sampling periods might contribute to such disparities, although a longer time series may improve resolution. 

In this study, we captured *P. juglandis* in 12-unit pheromone-baited funnel traps in California, USA, for almost three years (from 29 August 2011 to 2 June 2014). Traps were sampled weekly. We sought to develop and present multiple regression models that can explain weekly catches of *P. juglandis* with a comprehensive set of weather variables (i.e., precipitation, solar radiation, vapor pressure, air temperature, relative humidity, and wind speed) from this long-term study. Our objective was to create models that are simple to use, provide strong explanatory power, and are useful for estimating trends in trap catches of *P. juglandis*. Although the models are specific to California, we expected that knowledge of variables associated with the flight of *P. juglandis* may be useful in developing forecasting tools for this beetle as it invades new areas.

## 2. Materials and methods

### 2.1. Study Site, Flight Trapping, and Beetle Handling

The study site, flight trapping, and beetle handling techniques have been described elsewhere [15,16]. Briefly, the study site was a native riparian forest stand of northern California black walnut, *Juglans hindsii* (Jeps.) Jeps. *ex* R.E. Sm., Fremont’s cottonwood, *Populus fremontii* S. Wats., and valley oak, *Quercus lobata* Née, located along the north fork of Putah Creek in Davis (38°32’20.66’’ N, 121°44’21.42’’ W, approx. 16 m elev.) in Yolo Co., California, USA. Five twelve-unit black plastic multiple funnel traps were baited with the *P. juglandis* aggregation pheromone [29] and spaced at a distance greater than 50 m from each other at the study site. Traps were placed 3–5 m from the main stem of a *J. hindsii* tree and on top of a 3 m pole [30]. A collection cup with ~100 mL of ethanol-free, propylene glycol-based antifreeze was attached to each trap. 

Traps were emptied weekly at 0800 h of every Monday starting 7 November, 2011 to 7 May, 2012, and from 18 September, 2012 to 2 June, 2014. Insects were transported back to the laboratory, where total numbers of male and female *P. juglandis* were tabulated. In this work, we also included weekly *P. juglandis* catches from 29 August to 7 November, 2011 and 8 May to 17 September, 2012 from a previously published dataset [15]. In total, the complete dataset includes 142 weekly *P. juglandis* catches (pooled over the five traps) from 29 August, 2011 to 2 June, 2014. Trap catch data from two weeks (20 to 27 May, 2013, and 20 to 27 January, 2014, were missing) [16].

### 2.2. Nomenclature

In this project, we have used the original nomenclature for bark and ambrosia beetles (Coleoptera: Scolytidae) based on the argument presented in Wood [31] and a more extensive treatment of the issue developed by D.E. Bright in his third supplement to the world catalog of the Scolytidae and Platypodidae [2]. In essence, morphological and fossil evidence of adult scolytids support the family-level treatment, whereas similarity in scolytid and curculionid larval morphology supports a subfamily placement. As this issue is not entirely resolved, we prefer to take the more conservative approach of using the original nomenclature.

### 2.3. Weather Data

Daily weather data were obtained from the California Irrigation Management Information System, Department of Water Resources (CIMIS) website (http://www.cimis.water.ca.gov/). We utilized weather station #139, located approximately 100 m northwest of the trapping site. Weather data include daily precipitation (mm), solar radiation (W m^-2^), average vapor pressure (kPa), maximum air temperature (°C), minimum air temperature (°C), average air temperature (°C), maximum relative humidity (%), minimum relative humidity (%), average relative humidity (%), average wind speed (m s^-1^), and average soil temperature (°C). From these daily data, we generated weekly weather variables that might affect insect flight (Table 1).

### 2.4. Model Development

We approached model building to explain weekly catches of *P. juglandis* in two stages. First, we examined temporal dependence in the time series by using the autocorrelation function acf in the car package within *R* [32]. Insect catches collected over time generally showed a temporal pattern, suggesting that they might be autocorrelated at finer time scales [19]. Autocorrelation can bias influential tests associated with parameter estimates, so we removed autocorrelation by introducing lagged response variables as dependent variables as required (i.e., number of insects captured as a function of numbers captured in collection period one, two, etc., weeks previous). Second, we began including weather variables by using two different approaches. The weekly *P. juglandis* catches were regressed on the weather variables (Table 1), separately for males, females, and the total of males and females, by using both backward elimination and forward selection model-building techniques. A backward elimination procedure fitted the full model with the selected temporal terms and all weather variables, and then iteratively dropped those weather variables with the highest *p*-value one at a time until all remaining weather variables were significant at *α* = 0.05. To avoid or mitigate multicollinearity concerns, some highly correlated (*r* > 0.90) weather variables were excluded from the full model (see Table 1 for weather variables included in the full model in the backward elimination approach). Alternatively, a forward selection method started with a null model of previously selected temporal variables, and then added each weather variable one at a time so that separate models were created, containing the temporal terms and an individual weather variable. The model with the lowest Akaike Information Criterion (AIC) was then selected and formed the basis of the next step. All remaining weather variables were added one at a time to that model, creating a new set of models. Again, the best model was selected by using AIC. This process continued until a better model could not be identified.

For all models, *P. juglandis* catches and temporal variables were natural logarithm-transformed after an addition of one. We also calculated the partial *R*^2^ (coefficient of partial determination) for all variables included in the final models. This measure estimates the proportion of variation in trap catch that is explained with the addition of that variable to the model, assuming the other variables have already been added. As such, high numbers indicate that the variable is more “useful” for explaining variation in weekly *P. juglandis* trap catch. Coefficients of partial determination were obtained by the function *modelEffectSizes* in the package *lmSupport* in *R*.

Model assumptions such as the homoscedasticity and normality of residuals were checked by using graphical examination of residual plots. Lack of significant autocorrelation remaining in model residuals was verified with a Durbin–Watson test for the time series (package *car* in *R*).

## 3. Results

**Figure 1:**
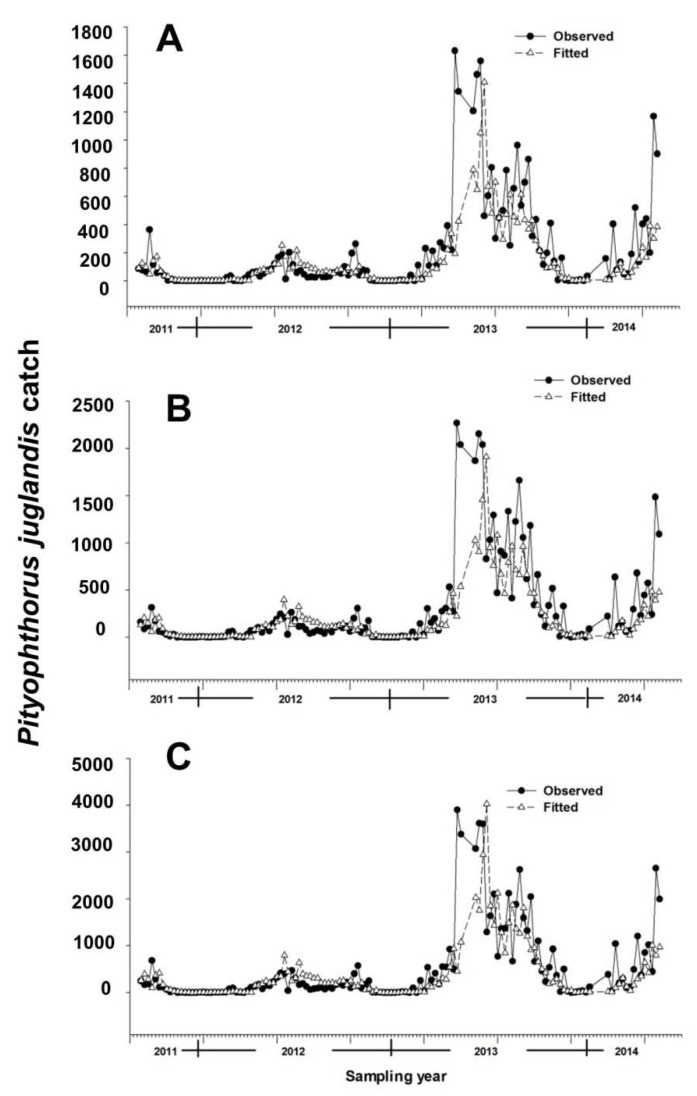
Observed and fitted weekly catches of *Pityophthorus juglandis* per trap (*N* = 5). (**A**) Male; (**B**) Female; and (**C**) Total. Equations for fitted models are given in Table 2. *Pityophthorus juglandis* was collected weekly from 29 August, 2011 to 2 June, 2014 in a native riparian forest stand of northern California black walnut, *Juglans hindsii*; Fremont’s cottonwood, *Populus fremontii*; and valley oak, *Quercus lobata*, located along the north fork of Putah Creek in Davis in Yolo Co., California, USA. Fitted data were extracted from models built on logarithm-transformed, and then exponent-transformed, data. Vertical bars in the x-axis separate years.

A total of 74,522 *P. juglandis* (44,011 females and 30,511 males) was collected during the 142 weeks. Between 2011 and 2014, *P*. *juglandis* generally initiated flight in late January and continued until late November (Figure 1). This seasonal flight could be divided approximately into three phases (emergence: January–March; primary flight: May–July; and secondary flight: September–October). The seasonal flight response to the male-produced aggregation pheromone was consistently female-biased (mean of 58.9% females). Diurnal flight followed a bimodal pattern with a minor peak in mid-morning and a major peak at dusk (76.4% were caught between 1800 and 2200 h) (see [16] for detailed analyses of seasonal and diurnal flight).

**Figure 2:**
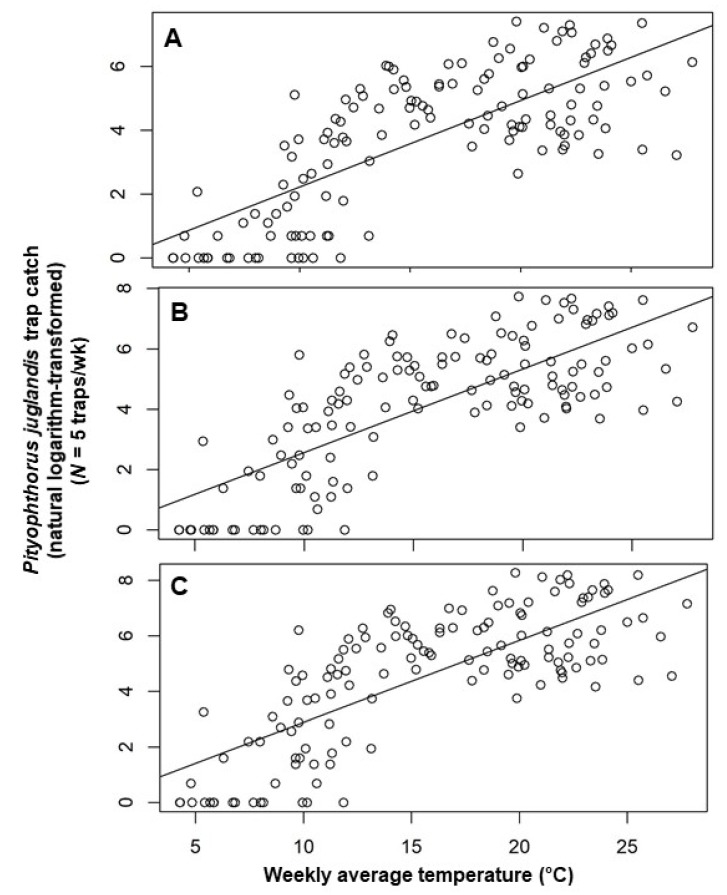
Scatter plot of weekly catches of *Pityophthorus juglandis* per trap (natural logarithm-transformed) against weekly average temperature (°C). (**A**) Male; (**B**) Female; and (**C**) Total. The straight line is the linear regression fitted by ordinary least square. *Pityophthorus juglandis* was collected weekly from 29 August, 2011 to 2 June, 2014 in a native riparian forest stand of northern California black walnut, *Juglans hindsii*; Fremont’s cottonwood, *Populus fremontii*; and valley oak, *Quercus lobata*, located along the north fork of Putah Creek in Davis in Yolo Co., California, USA.

**Figure 3:**
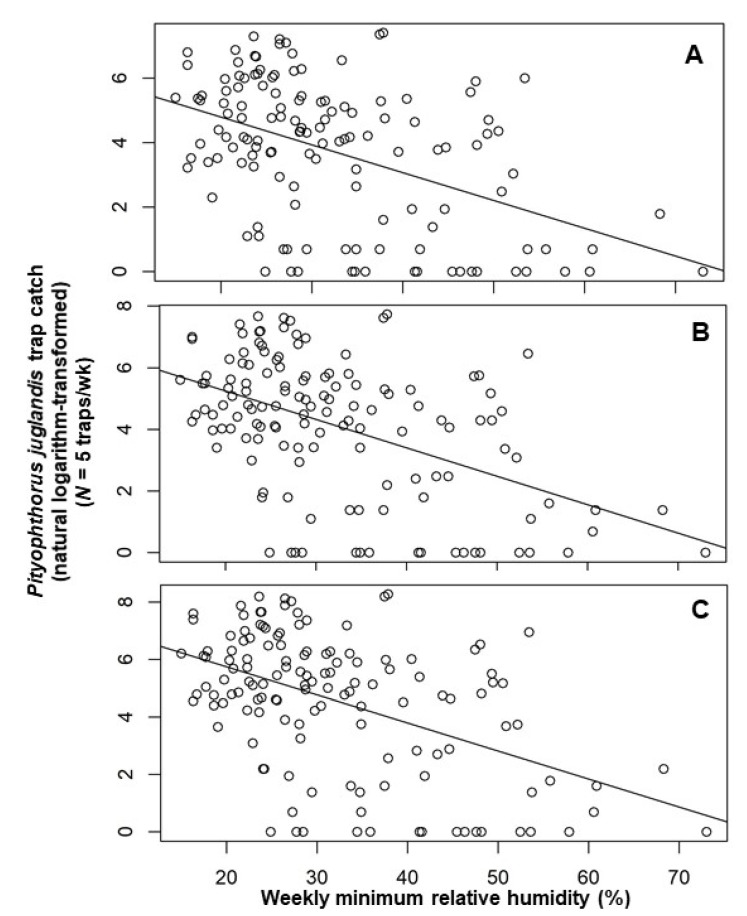
Scatter plot of weekly catches of *Pityophthorus juglandis* per trap (natural logarithm-transformed) against weekly average minimum relative humidity (%). (**A**) Male; (**B**) Female; and (**C**) Total. The straight line is the linear regression fitted by ordinary least square. *Pityophthorus juglandis* was collected weekly from 29 August, 2011 to 2 June, 2014 in a native riparian forest stand of northern California black walnut, *Juglans hindsii*; Fremont’s cottonwood, *Populus fremontii*; and valley oak, *Quercus lobata*, located along the north fork of Putah Creek in Davis in Yolo Co., California, USA.

Multiple regression models constructed by using a backward elimination procedure identified two temporal variables as important in explaining the number of beetles caught in a trap in any given week: the trap catches from each of the two preceding collection periods (Table 2). The inclusion of terms for mean weekly air temperature and mean minimum relative humidity also helped explain male, female, and total beetle catches (Table 2). After accounting for other terms in the models, an increment of 0.08 [=exp (0.08) − 1] males, 0.09 [=exp (0.09) − 1] females, or 0.11 ([=exp (0.11) − 1] total beetles were noted per degree Celsius increase in temperature (Table 2). *Pityophthorus juglandis* catches were correlated negatively with average minimum air relative humidity, with a decrease of 0.02 [=exp (0.02) − 1] beetles (male catches) or 0.03 [=exp (0.03) − 1] beetles (female and total catches) per one percent rise in humidity (Table 2). The catches of *P*. *juglandis* were correlated with mean air temperature and mean minimum humidity independently of other variables (Figure 2 and Figure 3).

The models fit the data well, particularly for weeks exhibiting catches of fewer than 1000 beetles, as 75%, 72%, and 73% (male, female, and total catches, respectively) of variances were explained by the variables included in the models (Table 2 and Figure 1). The partial *R*^2^ values between weather variables and *P. juglandis* catches, regardless of whether the catches were male, female, or total, were never more than 0.06 (Table 3). In contrast, the partial *R*^2^ values for the temporal variables ranged from 0.09 to 0.20 (Table 3). Thus, knowledge of previous week(s) trap catches is more useful than knowledge of weather patterns in predicting trap catches if both pieces of information are not available.

Utilization of a forward selection method resulted in multiple regression models with the same two temporal variables, but also terms for total solar radiation, average vapor pressure, maximum air temperature, average minimum air relative humidity, and average soil temperature. However, we found that average weekly air temperature was highly and positively correlated with total solar radiation (*r* = 0.88), average vapor pressure (*r* = 0.76), maximum air temperature (*r* = 0.96), and average soil temperature (*r* = 0.96). Thus, for simplicity, we do not further report on these models.

## 4. Discussion

One of the first steps in developing an integrated management plan for a new invasive species entering a new range is to delimit its activity and seasonal phenology. This work offers a simple method to describe the week-to-week flight activity of *P. juglandis* in California, but we expect it may be easily applied to forecast activity in other parts of its range. For example, the consistently high partial *R*^2^ values for temporal vs. weather variables, irrespective of model-fitting technique (Table 3), suggest that flight patterns may be predicted with moderate success in new areas by using previous week(s)’ captures, even before refinements have been made with the current week’s local weather data. The lineage of *P. juglandis* present at the Yolo Co., CA test site is most likely the so-called L1 lineage, which is the most widely represented (and has been the most invasive) in the USA. This ubiquity suggests a wider applicability of the models for flight prediction, but we realize that attempts to extend the models for predicting flight from northern California riparian habitats to other sites within or beyond California may benefit from additional site-specific weather variables.

Weekly *P. juglandis* catches in this study increased at a rate of approximately one beetle per degree Celsius with increasing weekly mean air temperature. That many insects actively regulate body temperature, in particular, thoracic temperature, by behavioral or physiological means before and during the onset of flight [33,34,35,36,37,38,39], indicates the existence of a lower and an upper threshold for flight [40]. *Pityophthorus juglandis* flight within a day generally increases with rising temperature when temperatures are between 11 °C (lower threshold) and 27 °C before decreasing to the cessation of flight at temperatures above 39 °C [16]. It is intuitive that insect flight increases with increasing temperature before temperature reaches an optimum. It has long been known that many insects, and particularly bark beetles, do not fly below a lower temperature threshold [37,41,42], and insects flying in cold temperatures need to make greater efforts to generate body temperature to maintain flight [43]. Some insects make metabolic preparations before flight that can take as long as 6 min [33]. Under lower temperatures, the capability or efficiency of the oscillatory flight of Asiatic rhinoceros beetle, *Oryctes rhinoceros* (L.) (Coleoptera: Scarabaeidae), is reduced [44]. Conversely, increasing temperature increases aerodynamic force production in the fruit fly, *Drosophila melanogaster* Meigen (Diptera: Drosophilidae) [45], and mechanical power output in the tobacco hawkmoth, *Manduca sexta* (L.) (Lepidoptera: Sphingidae) [46], probably by increasing wing-beat frequency, as shown in the American cockroach, *Periplaneta americana* (L.) (Blattodea: Blattidae) [21]. The maximum weekly mean air temperature in the study was 27.8 °C, which is near the optimal temperature at which *P. juglandis* flight peaks [16]. An alternative, but not mutually exclusive explanation for increased trap catches at warmer temperatures is simply a positive correlation between temperature and lure elution rate.

*Pityophthorus juglandis* catches were correlated negatively with mean minimum air relative humidity with a decrease of approximately one beetle per one percent humidity. The negative correlation between insect flight and humidity might be caused by the higher requirement of wing-beat frequency at a higher humidity than at a lower humidity. Wing-beats are metabolically costly [33,39]. *Periplaneta americana* beats its wings more frequently at 95% relative humidity than at 50% when the temperature falls between 27 and 35 °C [21]. The higher wing-beat frequency at a higher humidity could reflect greater efforts required to dissipate heat at higher vs. lower humidities [21]. Church [34], however, proved experimentally that heat loss by convection, not by evaporation (or water loss), is the single most important means of heat loss and accounts for 60% to 80% of heat loss in many insects. On the other hand, Juillet [20] postulated that insect flight response to various humidity levels is an adaptation to various niches. For example, the preference of wasps in the Ichneumonidae for high humidity coincides with their niches in forest habitats, whereas the preference of wasps in the Braconidae for low humidity coincides with their niches in more open agricultural settings. *Pityophthorus juglandis* colonizes declining or dying walnut trees that are probably under high- or low-moisture stress [4]. This might explain the decline in flight with increasing humidity for *P. juglandis* in this study. The flight capacity of *P. juglandis* without the intervention of wind is low: the maximum distance flown within 24 h is approximately 3.6 km [17]. Monte Carlo simulation indicated that only 1% of beetles would be able to fly farther than 2 km in a 5 d period; around 33% fly less than 100 m during 5 d [17]. As such, days with high humidity may limit *P. juglandis* flight and, for early detection of the beetle, it is advisable to set up traps prior to sunny and relatively dry days for optimal detection.

In summary, understanding when *P. juglandis* may be active in dispersal and host-finding may be valuable for the development of an integrated pest management program. Models considering trap captures from the previous two weeks, current mean air temperature, and current mean minimum air relative humidity fit the trap catch data well (particularly when catches were less than 1000 beetles). The proportions of the variances explained by the variables included in the model were 75%, 72%, and 73% for male, female, and total beetle catches, respectively (Table 1 and Figure. 1). These models facilitate our understanding of the environmental factors that dictate the flight of *P. juglandis*. Future steps to expand on this work may include using independent, long-term datasets reflecting various *P. juglandis* population densities at various locations and habitats to enhance model validation and to test the performance of the model.

## 5. Conclusions

Captures of *P. juglandis* increased with mean weekly air temperature and decreased with increasing mean minimum relative humidity in the current study, regardless whether models were developed for males, females, or both sexes pooled. *Pityophthorus juglandis* captured in each of the preceding two weeks contributed most to a current week’s catch. This suggests that the models developed in the current study site might be generalized to other locations.

## Figures and Tables

**Table 1:** Variables used to develop a flight model of weekly catches of walnut twig beetle (WTB), *Pityophthorus juglandis*, captured in pheromone-baited funnel traps, in California, USA^1^.

Variables	Explanation	Mean	Min	Max
WTB_M	Weekly male *P. juglandis* catches	216.0	0.0	2177.0
WTB_F	Weekly female *P. juglandis* catches	312.0	0.0	2805.0
WTB	Weekly *P. juglandis* catches, males and females pooled	529.0	0.0	4982.0
Lag 1	*P. juglandis* catches in the preceding collection	-	-	-
Lag 2	*P. juglandis* catches 2 weeks preceding the current collection	-	-	-
Mean_Prec	Average precipitation (mm) in the week	1.0	0.0	16.5
Days_Prec	Number of days with precipitation > 1 mm in the week	1.1	0.0	6.0
Mean_Sol	Average solar radiation (W m^-2^) in the week	195.8	47.6	343.9
Mean_Pres	Average vapor pressure (kPa) in the week	1.0	0.4	1.6
Max_Temp ^2^	Maximum air temperature (°C) in the week	23.9	10.6	37.0
Mean_Max_Temp ^2^	Average maximum air temperature (°C) in the week	27.4	12.7	40.5
Min_Temp ^2^	Minimum air temperature (°C) in the week	4.3	−7.2	14.1
Mean_Min_Temp ^2^	Average minimum air temperature (°C) in the week	7.8	−3.4	17.9
Mean_Temp	Average air temperature (°C) in the week	15.7	4.3	27.8
Max_Rel	Maximum air relative humidity (%) in the week	82.2	45.1	96.4
Mean_Max_Rel	Average maximum air relative humidity (%) in the week	91.3	70.0	97.0
Min_Rel	Minimum air relative humidity (%) in the week	32.2	13.4	73.0
Mean_Min_Rel ^2^	Average minimum air relative humidity (%) in the week	18.6	5.0	55.0
Mean_Rel	Average air relative humidity (%) in the week	53.6	23.3	90.0
Max_Wind	Maximum wind speed (m s^-1^) in the week	2.2	1.1	4.8
Min_Wind	Minimum wind speed (m s^-1^) in the week	1.1	0.7	2.1
Mean_Wind	Average wind speed (m s^-1^) in the week	1.5	0.9	2.6
Max_Soil_Temp ^2^	Maximum soil temperature (°C) in the week	16.9	6.2	26.5
Min_Soil_Temp ^2^	Minimum soil temperature (°C) in the week	15.5	5.7	25.3
Mean_Soil_Temp	Average soil temperature (°C) in the week	16.1	6.0	25.8

^1^*Pityophthorus juglandis* was collected weekly from 29 August, 2011 to 2 June, 2014 in a native riparian forest stand of northern California black walnut, *Juglans hindsii*; Fremont’s cottonwood, *Populus fremontii*; and valley oak, *Quercus lobata*, located along the north fork of Putah Creek in Davis in Yolo Co., California, USA. Daily weather data were obtained from weather station #139, located approximately 100 m northwest of the trapping site, from the California Irrigation Management Information System, Department of Water Resources (CIMIS) website (http://www.cimis.water.ca.gov/). See Supplementary for correlation coefficients between the listed weather variables. ^2^ These weather variables were excluded from the full model for the backward elimination approach.

**Table 2:** Multiple regression models developed for 142 weekly trap catches of walnut twig beetle (WTB), *Pityophthorus juglandis* ^1.^

WTB Catches ^2^	Regression Variables					Model Summary			
	Intercept (±SE)	Temporal (±SE) ^3^		Mean (±SE) Weekly Air Temperature (°C)	Mean (±SE) Minimum Air Relative Humidity (%)	*R* ^2^_adj_	*F*	*df*	*p*
		Lag 1	Lag 2						
Male	0.85 ± 0.55 (*p* = 0.121)	0.32 ± 0.08 (*p* < 0.001)	0.38 ± 0.08 (*p* < 0.001)	0.07 ± 0.03 (*p* = 0.012)	−0.02 ± 0.03 (*p* = 0.018)	0.75	101.00	4, 130	<0.01
Female	1.22 ± 0.58 (*p* = 0.038)	0.28 ± 0.08 (*p* < 0.001)	0.36 ± 0.08 (*p* < 0.001)	0.08 ± 0.03 (*p* = 0.006)	−0.03 ± 0.01 (*p* = 0.005)	0.72	87.91	4, 130	<0.01
Total ^4^	1.35 ± 0.61 (*p* = 0.028)	0.29 ± 0.08 (*p* < 0.001)	0.36 ± 0.08 (*p* < 0.001)	0.08 ± 0.03 (*p* = 0.008)	−0.03 ± 0.01 (*p* = 0.005)	0.73	91.36	4, 130	<0.01

^1^*Pityophthorus juglandis* was collected weekly from 29 August, 2011 to 2 June, 2014 in a native riparian forest stand of northern California black walnut, *Juglans hindsii*; Fremont’s cottonwood, *Populus fremontii*; and valley oak, *Quercus lobata*, located along the north fork of Putah Creek in Davis in Yolo Co., California, USA. ^2^ Response variable transformed ln(y+1). ^3^ Lag 1: Catch of *P. juglandis* during the preceding week; Lag 2: Catch of *P. juglandis* during the week at two weeks preceding the current week ^4^ Males + females.

**Table 3:** Partial *R*^2^ between independent and dependent variables in multiple regression models (Table 2) developed for 142 weekly trap catches of walnut twig beetle (WTB), *Pityophthorus juglandis* ^1.^

WTB Catches ^2^	Regression Variables				
	Intercept	Temporal Variables ^3^		Mean Weekly Air Temperature (°C)	Mean Minimum Air Relative Humidity (%)
		Lag 1	Lag 2		
Male	0.02	0.12	0.16	0.03	0.03
Female	0.03	0.09	0.14	0.06	0.06
Total^4^	0.04	0.10	0.15	0.05	0.06

^1^*Pityophthorus juglandis* was collected weekly from 29 August, 2011 to 2 June, 2014 in a native riparian forest stand of northern California black walnut, *Juglans hindsii*; Fremont’s cottonwood, *Populus fremontii*; and valley oak, *Quercus lobata* located along the north fork of Putah Creek in Davis in Yolo Co., California, USA. ^2^ Response variable transformed ln(y+1). ^3^ Lag 1: Catch of *P. juglandis* during the preceding week; Lag 2: Catch of *P. juglandis* during the week at two weeks preceding the current week. ^4^ Males + females.

## Dedication

Y.C. and B.H.A. dedicate this manuscript to S.J.S. for his life-long contributions to the chemical ecology and management of forest pests. Tragically, Steve passed away in the middle of the submission of this work.
